# A Common Variant of *ASAP1* Is Associated with Tuberculosis Susceptibility in the Han Chinese Population

**DOI:** 10.1155/2019/7945429

**Published:** 2019-04-08

**Authors:** Cheng Chen, Qi Zhao, Yan Shao, Yan Li, Honghuan Song, Guoli Li, Limei Zhu, Wei Lu, Biao Xu

**Affiliations:** ^1^Department of Chronic Communicable Disease, Center for Disease Control and Prevention of Jiangsu Province, Nanjing, China; ^2^School of Public Health, Fudan University, Shanghai, China; ^3^Key Lab of Health Technology Assessment, National Health Commission of the People's Republic of China, Fudan University, Shanghai, China; ^4^Department of Public Health Sciences (Global Health/IHCAR), Karolinska Institutet, Stockholm, Sweden

## Abstract

**Background:**

*ASAP1* (also known as AMAP1 or DDEF1) encodes an Arf GTPase-activating protein (Arf GAP), a multifunctional scaffold protein that induces hydrolysis of GTP bound to the ADP-ribosylation factor (Arf) family GTP-binding proteins. Reduction of *ASAP1* expression in vitro was related to suppression of cell migration and invasiveness. The genetic variant rs4733781 of the *ASAP1* gene was revealed as a significant locus for tuberculosis (TB) susceptibility, but the results still need to be validated.

**Methods:**

Blood samples from a total of 1914 active TB and healthy controls (HC) were collected to evaluate rs4733781 and the risk of TB. Meanwhile, a total of 48 noninfected HC, latent TB-infected (LTBI) controls, and active TB were collected to assay *ASAP1* expression difference among the three groups. The QuantiFERON-TB Gold In-Tube was adopted to identify noninfected HC and LTBI.

**Results:**

The genetic variant of rs4733781 was found to be significantly associated with TB, and the A allele of rs4733781 (C>A) was 0.38 and 0.43 among TB cases and HC, respectively (*P* = 0.0035). Meanwhile, the peripheral blood monocyte RNA fold changes for the *ASAP1* gene among the 16 HC, 16 LTBI, and 16 active TB were 1.088 ± 0.4919, 2.237 ± 0.6505, and 10.12 ± 10.98 (*F* = 9.559, *P* = 0.0003), respectively, and the expression of *ASAP1* was increased by 2.06-fold (*P* < 0.0001) and 9.30-fold (*P* < 0.0052) for LTBI and active TB, when compared to the HC.

**Conclusions:**

Our data indicated that the A allele of rs4733781 for the *ASAP1* gene was in association with a decreased risk of TB. But not only that, the overexpression of the *ASAP1* gene among LTBI and TB was related to the progression of TB, which further implies that the expression of *ASAP1* would be a potential biomarker for LTBI and TB diagnoses.

## 1. Introduction

According to the recent global tuberculosis (TB) report, TB is the ninth leading cause of death worldwide and the leading cause of a single infectious agent, ranking above HIV/AIDS [1].

Meanwhile, a recent cohort study with large-scale population demonstrated a high latent tuberculosis infection (LTBI) rate of around 20% in China [2]. However, not all of those infected individuals will proceed into TB, and it was estimated that around 10% of the LTBI population will finally develop TB in their whole lifetime [3].

As we know, diabetes [4], receiving anti-TNF*α* drugs [5], and HIV infection are commonly considered the risk factors for TB [6, 7]. Nevertheless, the previous study has provided important clues that the host genetics was indispensable for the development of TB [8]. Previous studies had reported that genetic variation of cytokines would be involved in the susceptibility to TB, such as interleukin 6 [9], interleukin 10 [10], and interleukin 17 [11]. Meanwhile, genetic polymorphism of the vitamin D receptor was also reported in association with TB [12].

In recent years, the whole genome-wide association studies (GWAS) revealed some significant loci which demonstrated high correlation with TB [13, 14]. Meanwhile, the peripheral blood gene expression signatures for distinguishing LTBI and active TB patients would be helpful in predicting the risk of TB and even in monitoring the efficacy of the anti-TB treatment process [15, 16].

*ASAP1* (also known as AMAP1 or DDEF1) encodes an Arf GTPase-activating protein (Arf GAP), a multifunctional scaffold protein that induces hydrolysis of GTP bound to the ADP-ribosylation factor (Arf) family GTP-binding proteins [17]. Reduction of *ASAP1* expression in vitro was connected with suppression of cell migration and invasiveness [18]. Overexpression of *ASAP1* has been associated with metastasis in cancers [19, 20].

The genetic variants of *ASAP1* were firstly reported in association with TB susceptibility by Curtis et al. in 2015, and the functional genetic variant rs4733781 was revealed as the most significant locus for TB susceptibility [21]. Thereafter, only two studies conducted by Hu et al. and Miao et al., who had tried to validate this significant finding in Chinese Han population, finally reached negative results [22, 23]. Recently, rs4733781 was found in association with TB in Xinjiang Muslim population again [24]. In order to identify the effect of this genetic variant on TB susceptibility, we conducted this case-control study with large sample size in a Han Chinese population. Moreover, we will evaluate the expression of *ASAP1* mRNA in TB cases and the controls to further support the functional role of *ASAP1* in the development of TB.

## 2. Methods

### 2.1. The Sample for rs4733781 Genotyping

In total, 957 new incident TB cases and 957 healthy controls (HC) were recruited for this case-control study, and all the participants were belonging to Chinese Han ethnicity. All enrolled cases were bacteriologically confirmed by Lowenstein-Jensen (LJ) culture, and the M. tuberculosis was identified by the p-nitrobenzoic acid (PNB) method; TB cases were collected on July 1st, 2013, to December 31st, 2014, in Danyang County and Nanjing City of Jiangsu Province. Meanwhile, the HC were recruited from two communities of Danyang County. All control candidates received the X-ray examination. Sputum culture was provided if the controls reported TB-like clinical symptoms. Only participants with normal X-ray manifestation, negative LJ culture if tested, and no comorbidity with other infectious diseases (such as HIV/AIDS and Hepatitis B virus) were eligible for inclusion. Controls were 1 : 1 matched to the cases by age (±5 years) and gender. The interferon-gamma release assay (QuantiFERON-TB Gold In-Tube [QFT; Qiagen, Valencia, CA, USA]) was used to test the latent TB infection (LTBI) status of the HC. The QFT was performed according to the instructions provided by Qiagen [25].

### 2.2. Samples for the *ASAP1* Expression Assay

In this study, we will further evaluate the *ASAP1* gene expressions in individuals free of TB infection, with LTBI, and of active TB. Individuals free of TB infection and with LTBI were screened from a health examination population in Jurong County in 2016. The T-SPOT assay was adopted to detect the status of LTBI, and the assay procedure can be referred to the instruction by Oxford Immunotec [25]. Meanwhile, all the participants were tested by X-ray examination and met the normal manifestation standards for qualified enrollment. Finally, sixteen LTBI participants and sixteen noninfected HC were included (Table 1). Culture-confirmed active TB cases without initiating treatment were enrolled from the Chest Hospital of Nanjing from June to August of 2015, and 16 TB cases were finally included for the *ASAP1* expression assay (Table 1). The whole blood was obtained for the *ASAP1* expression assay from each individual free of TB infection, with LTBI, and of active TB. All experimental protocols in this study were approved by the Institutional Review Board of Center for Disease Control and Prevention of Jiangsu Province, and written informed consent was obtained from each participant before the study.

### 2.3. Genomic DNA and mRNA Extractions

Genomic DNA was extracted from the whole blood by proteinase K digestion and followed by classical phenol/chloroform extraction and ethanol precipitation. Genomic DNA was dissolved in TE buffer and diluted to 20 ng/*μ*L for use. Genomic mRNA was extracted from the PBMCs by the TRIzol® LS reagent (Ambion®, Carlsbad, CA, USA), and the isolation procedure was referred to the manufacturer's instructions. The genomic mRNA was dissolved with RNase-free water and diluted to 50 ng/*μ*L for use. The quality of the extracted genomic DNA and mRNA was quantitated spectrophotometrically by the NanoDrop 2000 instrument (Thermo Fisher Scientific, Waltham, MA, USA).

### 2.4. Genotyping of rs4733781

rs4733781 was genotyped by the TaqMan® SNP Genotyping Assay. The assay probe was commercially provided by Thermo Fisher Scientific (MA, USA). The assay ID was C__28031183_10. SNP genotyping was performed on the QuantStudio™ Dx Real-Time PCR instrument (Applied Biosystems, Foster City, CA, USA). The PCR reaction program was as follows: step 1: 60°C for 30 seconds; step 2: 95°C for 10 minutes; step 3: by 40 cycles, 95°C for 15 seconds and 60°C for 1 minute; and step 4: 60°C for 30 seconds.

### 2.5. mRNA Reverse Transcription

High-Capacity cDNA Reverse Transcription Kits (Applied Biosystems, Foster City, CA, USA) were used for the whole genomic mRNA reverse transcription. The reverse transcription procedure was conducted according to the instructions provided by the manufacturer. Meanwhile, primers for the reference gene GAPDH were designed to assay the quantity of the cDNAs (forward-GAA ATC CCA TCA CCA TCT TCC AGG, reverse-GAG CCC CAG CCT TCT CCA TG), and the length of the amplified GAPDH gene fragment was 120 base pairs.

### 2.6. Gene Expression Assay

2X TaqMan® Universal Master Mix II (Applied Biosystems, Foster City, CA, USA) with a reaction volume of 10 *μ*L was used to quantitate the gene expressions. Each reaction contains 5 *μ*L 2X TaqMan® Universal Master Mix II, 0.5 *μ*L TaqMan® Gene Expression Assay Mix, 3.5 *μ*L RNase-free water, and 1 *μ*L cDNA template. The TaqMan® Gene Expression Assay MIX ID for *ASAP1* genes was Hs00393663_m1. 18S was adopted as the reference gene. The gene expression assay was performed on the QuantStudio™ Dx Real-Time PCR instrument (Applied Biosystems, Foster City, CA, USA), and the program was as follows: step 1: 95°C for 10 minutes and step 2: by 40 cycles, 95°C for 10 seconds and 60°C for 1 minute.

### 2.7. GEO Data Analysis for *ASAP1* Expression

Public gene expression microarray data (NCBI GEO https://www.ncbi.nlm.nih.gov/gds/) were employed to evaluate *ASAP1* expression differences among HC, LTBI, and TB. Only peripheral whole blood samples were chosen for the *ASAP1* expression assay. Three datasets (GSE19491 [26], GSE37250 [27], and GSE42834 [28]) were selected to evaluate *ASAP1* expression differences. The log-transformed fold change values for *ASAP1* were extracted for between-group comparison.

### 2.8. Statistics

The unpaired Student *t*-test and one-way ANOVA were applied to numerical variables, whereas the differences in categorical variables were tested by the *χ*^2^ test. Hardy-Weinberg equilibrium (HWE) was assessed by the Pearson *χ*^2^ test. The strength of associations between genotypes and TB was estimated by odds ratio (OR) and its 95% confidence interval (95% CI) through univariate and multivariate logistic regression analyses adjusted by age and gender. Online software GEO2R was applied to test *ASAP1* expression among different groups. The Benjamini and Hochberg (false discovery rate) was adopted for the multiple comparison correction. The value of fold change (FC) was calculated for showing the fluctuation of the gene expression among different groups. *P* value of less than 0.05 was considered statistically significant. All of the analyses were performed by SAS 9.3 software (SAS Institute Inc., Cary, NC, USA).

## 3. Results

A total of 1914 participants were finally recruited with a 1 : 1 ratio between the cases and controls to evaluate the association between rs4733781 and the risk of TB. Genotyping was failed in 9 samples (3 in the cases and 6 in the controls), so a total of 1905 samples were used for the analysis. The mean age was 53.15 ± 17.05 years for TB cases and 48.78 ± 17.54 years for the HC (*P* < 0.01 by the unpaired Student *t*-test for the two groups). Meanwhile, male cases constituted 71.9% (686/954) of the total TB cases. The QFT test was conducted on the peripheral blood sample for each HC to assay the status of LTBI, and 22.5% (215/951) of the HC were detected as LTBI.

As shown in Table 2, rs4733781, located within the intron of *ASAP1*, was found in significant association with TB. The heterozygote CA genotype was found in significant association with TB (OR = 0.81, 95% CI 0.66-0.99, *P* = 0.0373), and the mutant homozygote AA was associated with a decreased risk of TB (OR = 0.68, 95% CI 0.51-0.89, *P* = 0.0059). The dominant model of rs4733781 (CA+AA vs. CC) was also found in statistically significant association with TB (OR = 0.78, 95% CI 0.64-0.94, *P* = 0.0086). When TB cases were further compared with IGRA positive controls, the association between homozygote AA and TB was not significant (OR = 0.82, 95% CI 0.52-1.28, *P* = 0.383). However, the homozygote AA genotype of rs4733781 demonstrated a statistically significant association with TB by an OR of 0.64 among TB cases and IGRA negative controls (95% CI 0.47-0.86, *P* = 0.0034). The distribution of rs4733781 genotypes among IGRA-positive and IGRA-negative controls demonstrated no difference (Table 3).

**Figure 1:**
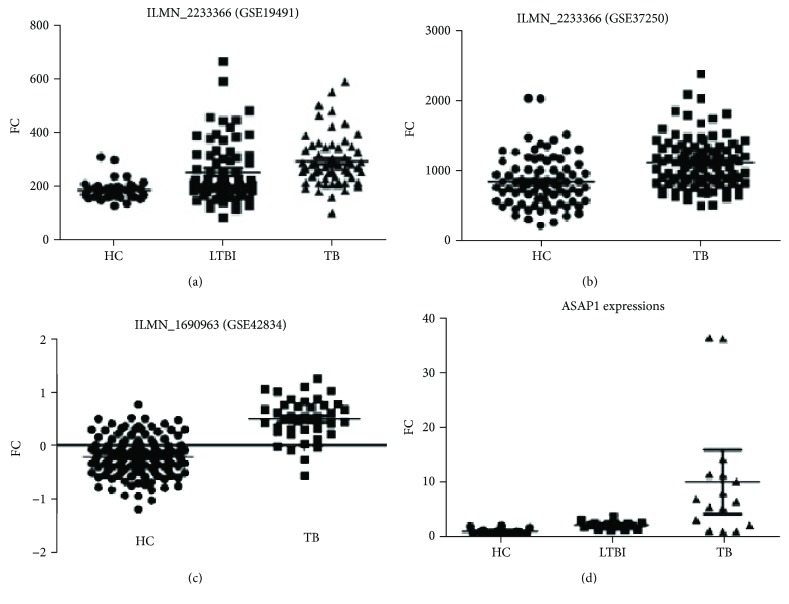
*ASAP1* expression difference among HC, LTBI, and TB. GSE19491 platform: Illumina HumanHT-12 V3.0 expression beadchip. GSE37250 platform: Illumina HumanHT-12 V4.0 expression beadchip. GSE42834 platform: Illumina HumanHT-12 V4.0 expression beadchip.

Three NCBI GEO datasets, GSE19491, GSE37250, and GSE42834, were chosen to test *ASAP1* expression difference among HC, LTBI, and TB, and all the enrolled participants were HIV negative. The whole blood monocyte cells were used to assay *ASAP1* expression for those three datasets. For GSE19491, *ASAP1* expressions among 36 HC, 69 LTBI, and 61 TB showed significant difference (Figure 1(a), *F* = 14.51, *P* < 0.0001). Meanwhile, *ASAP1* expression among LTBI was higher than HC (*t* = 3.382, *P* = 0.001), and *ASAP1* expression among the TB group was significantly higher than LTBI (*t* = 2.216, *P* = 0.0285). For GSE37250, only 83 LTBI and 97 active TB were included, and *ASAP1* demonstrated a higher expression among the TB group than LTBI (Figure 1(b), *t* = 4.879, *P* < 0.0001). The GSE42834 dataset only contained 118 HC and 40 TB, and *ASAP1* expressions in TB groups were significantly higher than HC as well (Figure 1(c), *t* = 10.04, *P* < 0.0001). We also included 16 HC, 16 LTBI, and 16 active TB in our study to evaluate *ASAP1* expressions, and we found that *ASAP1* expression was increased from HC to LTBI and much higher in TB (Figure 1(d)). The FC of *ASAP1* expression among the HC, LTBI, and TB were 1.088 ± 0.4919, 2.237 ± 0.6505, and 10.12 ± 10.98 (*F* = 9.559, *P* = 0.0003), respectively, and the expression of *ASAP1* was increased by 2.06-fold (*P* < 0.0001) and 9.30-fold (*P* < 0.0052) for LTBI and active TB, when compared to the HC.

## 4. Discussion

In this case-control study, genetic variant rs4733781 of the *ASAP1* gene was evaluated for association with the risk of TB, and rs4733781 was found in significant association with a low risk of TB in the Han Chinese population. The expression of *ASAP1* was increased among LTBI and much higher among TB patients.

SNP rs4733781, located in the intron region of *ASAP1*, was first found in association with TB risk by Curtis and colleagues in a Russian population [21]. However, the minor allele of rs4733781 in Curtis's study was C allele, which was different in our study, and Curtis and colleagues found that the C allele was in association with a decreased risk of TB. Not only that, Curtis and colleagues conducted a validation study in the African population, and they found that the C allele frequency in African population was much lower than in Russian, and the association between C allele and TB risk only reached a marginal significance.

In our study, the minor allele of rs4733781 was A allele, and it was found in significant association with a decreased risk of TB. Based on the QFT test, the control group was classified into HC and LTBI, the subgroups further evaluated their relationship with TB, and we found that A allele was significantly associated with TB between IGRA-negative controls and TB, except among IGRA-positive controls and TB. A previous study conducted in China explored rs4733781 and TB among the west Han Chinese and Tibetan populations [23], no significant effect of rs4733781 was found on TB, and the A allele frequencies were all above 50%, which was in reverse compared to our findings. The HapMap Han Chinese Being (HCB) data showed that the A allele of rs4733781 was 0.36, which was similar to our study. Another study conducted by Wang and colleagues in Xinjiang Muslim population found that the A allele was associated with a decreased risk of TB in China [24]. However, the minor frequency of rs4733781 in Xinjiang Muslim population was C allele, rather than A allele, which was different from our study, and this difference might be contributed to the different ethnic background. Nevertheless, Wang's study demonstrated the same effect of rs4733781 in association with TB. The minor allele of rs4733781 (A/C alleles) was different between African population and Asian population. Both minor allele A of rs4733781 in Chinese Han population and minor allele C of rs4733781 in African population showed significant association with a decreased risk of TB. Thus, we postulated that the two alleles, which represented a marker, would relate to other functional genes that are involved in the pathogenesis of TB susceptibility rather than directly involved in the pathogenesis of TB.

The reduction of *ASAP1* suppresses cell migration in vitro, and overexpression of *ASAP1* has been associated with metastasis in cancer [20].

Macrophages and dendritic cells (DCs) play an important role in TB pathogenesis, especially in the initial stage after *Mycobacterium* tuberculosis infection. DCs are important in the adaptive immunity initiation, but *Mycobacterium* tuberculosis is known to inhibit migration as well as other functions of DCs, as a result of a delayed adaptive immunity after *M*. tuberculosis infection [29, 30]. Just as Curtis and colleagues reported, the expression of *ASAP1* was lower among *Mycobacterium* bovis BCG-infected DCs when compared to noninfected DCs. It provided evidence to support that *Mycobacterium* tuberculosis infection delays adaptive immunity, and it seems that lower expression of *ASAP1* might be involved in the delayed adaptive immunity.

In our study, we found *ASAP1* expression was high among the LTBI group and much higher in TB patients, which indicated that *ASAP1* expression was increased after *Mycobacterium* tuberculosis infection, especially in TB disease status. We assayed *ASAP1* expression in PBMCs, which was different from DCs. Curtis and colleagues have not detected *ASAP1* expression among lymphocytes and monocytes after the infection of *Mycobacterium bovis* BCG, except DCs. Thus, based on Curtis' results, *ASAP1* expression might be decreased in the early stage of *Mycobacterium bovis* BCG infection. However, after DCs migrated to the lymph nodes, and T-cells being activated after adaptive immunity, we postulated that the expression of *ASAP1* would be stimulated by T-cell-based immunity during tuberculosis disease progression.

In our study, the expression of *ASAP1* was assayed from PBMCs, in which the expression of *ASAP1* might be different from the DCs. Nevertheless, according to a recent study by Zak et al., whole blood was collected directly into PAXgene blood RNA tubes for evaluation of the whole blood RNA signature changes from free of *Mycobacterium tuberculosis* infection to infection status and TB disease, and the whole blood-based signatures would be more feasible in predicting progression to active TB disease [15].

In conclusion, our study evaluated SNP rs4733781 on TB risk in a Han Chinese population, and the minor allele of rs4733781 was significantly associated with a decreased risk of TB; the expression of *ASAP1* was increased among LTBI and much higher among TB patients when compared to noninfected HC.

## Figures and Tables

**Table 1:** Basic characteristics of participants free of TB infection, with LTBI, and of active TB for the *ASAP1* expression assay.

Participants	Sample numbers	Ages	Gender	Clinical diagnosis
Noninfected HC	16	36 ± 5.94 (29~49)	Male: 3	X-Ray negative, no symptoms of TB, and no other infectious diseases and history of TB reported
			Female: 13	
Latent TB-infected HC	16	40.75 ± 7.34 (30~56)	Male: 2	X-Ray negative, no symptoms of TB, and no other infectious diseases and history of TB reported
			Female: 14	
Active TB	16	41.88 ± 20.60 (16~82)	Male: 10	Culture confirmed, all with clinical symptoms and X-ray abnormality of TB
			Female: 6	

HC: healthy controls; TB: tuberculosis.

**Table 2:** The genotype distribution of rs4733781 among tuberculosis cases and controls.

SNP rs4733781	Cases *n* (%)	Controls *n* (%)	^∗^OR (95% CI)	*P*	IGRA-positive controls *n* (%)	^∗^OR (95% CI)	*P*	IGRA-negative controls *n* (%)	^∗^OR (95% CI)	*P*
	954	951			215			736		
CC	361 (37.8)	304 (32.0)	1.00 (reference)		78 (36.3)	1.00 (reference)		226 (30.7)	1.00 (reference)	
CA	461 (48.3)	483 (50.8)	0.81 (0.66-0.99)	0.0373	102 (47.4)	0.98 (0.71-1.35)	0.8881	381 (51.8)	0.76 (0.61-0.95)	0.0159
AA	132 (13.8)	164 (17.2)	0.68 (0.51-0.89)	0.0059	35 (16.2)	0.82 (0.52-1.28)	0.382	129 (17.5)	0.64 (0.47-0.89)	0.0034
CA+AA	593 (62.2)	647 (68.0)	0.78 (0.64-0.94)	0.0086	137 (63.7)	0.94 (0.69-1.28)	0.677	510 (69.3)	0.73 (0.60-0.90)	0.0033
MAF	0.38	0.43								
HWE		0.24								

^∗^Adjusted by age and gender. HWE: Hardy-Weinberg equilibrium; IGRA: interferon-gamma release assay.

**Table 3:** Comparison of rs4733781 genotype distributions among IGRA-positive and IGRA-negative controls.

SNP rs4733781	IGRA-negative controls *n* (%)	IGRA-positive controls *n* (%)	^∗^OR (95% CI)	*P*
	736	215		
CC	226 (30.7)	78 (36.3)	1.00 (reference)	
CA	381 (51.8)	102 (47.4)	0.75 (0.53-1.06)	0.1007
AA	129 (17.5)	35 (16.3)	0.82 (0.51-1.31)	0.3939
CA+AA	510 (69.3)	137 (63.7)	0.76 (0.55-1.06)	0.1082

## Data Availability

The data used to support the findings of this study are available from the corresponding author upon request.
